# Factors Related to Perceived Stigma in Parents of Children and Adolescents in Outpatient Mental Healthcare

**DOI:** 10.3390/ijerph191912767

**Published:** 2022-10-06

**Authors:** Halewijn M. Drent, Barbara van den Hoofdakker, Jan K. Buitelaar, Pieter J. Hoekstra, Andrea Dietrich

**Affiliations:** 1 Department of Child and Adolescent Psychiatry, University Medical Center Groningen, University of Groningen, 9723 HE Groningen, The Netherlands; 2 Accare Child Study Center, 9723 HE Groningen, The Netherlands; 3 Department of Cognitive Neuroscience, Radboud University Medical Center, Donders Institute for Brain Cognition and Behaviour, 6525 AJ Nijmegen, The Netherlands

**Keywords:** adolescents, affiliate stigma, behavioral problems, children, child and adolescent psychiatry, emotional problems, outpatient mental healthcare, parents, perceived public stigma, predictors

## Abstract

Little is known about factors contributing to perceived stigma in parents of children and adolescents with behavioral and emotional problems in outpatient mental healthcare. We aimed to identify the most relevant factors related to perceived parental stigma using least absolute shrinkage and selection operator (LASSO) regression including a broad range of factors across six domains: (1) child characteristics, (2) characteristics of the primary parent, (3) parenting and family characteristics, (4) treatment-related characteristics, (5) sociodemographic characteristics, and (6) social–environmental characteristics. We adapted the Parents’ Perceived Stigma of Service Seeking scale to measure perceived public stigma and affiliate stigma in 312 parents (87.8% mothers) during the first treatment year after referral to an outpatient child and adolescent clinic. We found that the six domains, including 45 individual factors, explained 34.0% of perceived public stigma and 19.7% of affiliate stigma. Child and social–environmental characteristics (social relations) explained the most deviance in public stigma, followed by parental factors. The strongest factors were more severe problems of the child (especially callous–unemotional traits and internalizing problems), mental healthcare use of the parent, and lower perceived parenting competence. The only relevant factor for affiliate stigma was lower perceived parenting competence. Our study points to the multifactorial nature of perceived stigma and supports that parents’ perceived public stigma is susceptible to social influences, while affiliate stigma relates to parents’ self-evaluation. Increasing parents’ perceived parenting competence may help mitigate perceived stigma. Future studies should explore how stigma relates to treatment outcomes.

## 1. Introduction

Many parents of children with a mental health problem experience stigma [1,2,3]. Stigma can be defined as an attribute of an individual who is socially discredited by the larger public [4]. There are two important types of stigma in mental healthcare related to parents’ perceptions, (i) perceived public stigma, which refers to the awareness of stereotypical beliefs held by the larger public about mental illnesses or using mental health services and (ii) affiliate stigma (i.e., self-stigma of family members), which involves applying such negative stereotypes about stigmatized individuals to oneself, involving feelings of shame, embarrassment, or belittlement [5,6,7,8,9].

Stigma is increasingly being recognized as an important factor in child and adolescent mental healthcare, in which parents usually play an important role [10,11,12]. Stigma perceived by parents may not only act as a potential barrier for entering mental healthcare for their child [10,13,14], but may also be an important factor during treatment. Stigma may be associated with lower engagement in care, lower treatment adherence and efficacy, or earlier dropout, thus depriving parents and families of care they need [1,5,8,12,15,16]. It has been shown that parents were more likely to be involved in the treatment of their child if there was less stigma related to the treatment [5,7,10]. Perceived stigma may have negative consequences on parents’ affect, cognitions, and behavior; this includes low self-esteem, distress, or depression [17,18,19,20,21]; shame, self-blame, or guilt; secrecy and avoidance; feelings of isolation and rejection [2,3,22]; a poorer parent–child relationship [23] or poorer parental health [24]; all factors that may hamper treatment [5,9,12].

Perceived stigma has been related to a variety of factors suggesting a multifactorial nature [25]; perceived public stigma may particularly be affected by the social environment and affiliate stigma by parental factors relating to negative self-evaluation. However, to date, still little is known about the factors that may contribute to parents’ perceived stigma regarding having a child with emotional and/or behavioral problems treated in an outpatient mental health care setting, and, particularly, which are the most important factors [10,26,27,28]. Typically, individual studies have focused on a restricted number of factors and on children with specific mental disorders in isolation (such as autism spectrum disorder (ASD) or attention-deficit/hyperactivity disorder (ADHD), often including those with more severe problems [2,3,12,25], or on children with intellectual disabilities [19,29] or from rural community samples [30].

In earlier studies, higher perceived public stigma or affiliate stigma in parents of a child with mental health problems were found to be related to (1) child factors, such as more severe problem behavior of the child [31], (2) parental and family factors, such as a lower self-esteem [21], the sense of parenting competence [6], lower parental personal wellbeing [29,32], and higher parental stress [33], as well as lower family cohesion [21,34] and (3) social–environmental factors, such as less social support [19,32,35]. There are notable limitations of the current literature, with a lack of large samples including a variety of potential factors and the use of quantitative methods. While affiliate stigma has been studied most, risk factors of higher perceived public stigma in parents of a child with mental health problems are less well known. Of particular interest are factors that are potentially malleable in order to reduce or prevent parental perceived stigma, eventually aiding in the child’s treatment.

To summarize, currently, there is a dearth of literature on factors influencing parents’ perceived stigma and affiliate stigma in relation to their children’s emotional and/or behavioral problems. In the current study, we aimed to better understand which parents are more vulnerable to perceived public stigma and affiliate stigma during the treatment trajectory of their child in outpatient mental healthcare and to identify the most important contributors to parents’ perceived stigma. We therefore explored a broad set of factors, many of which may be a target of preventive strategies in relation to both types of stigma. We did this in a large sample of parents of children and adolescents who had been referred to one of the participating outpatient child and adolescent mental healthcare clinics and who had been under there care for one year. Factors included (1) child characteristics, (2) parental characteristics, (3) parenting and family characteristics, (4) treatment-related characteristics, (5) sociodemographic characteristics, and (6) social–environmental characteristics. The wide selection of predictor variables represents a synthesis of factors from previous research on public and affiliate stigma, extended by potential risk factors relevant to child and adolescent mental health, and should be regarded as both a confirmatory as well as a hypothesis-generating approach. We used least absolute shrinkage and selection operator regression (LASSO, [36]), which is particularly suited to identifying the most relevant factors among many potential variables. Our general hypothesis was that general risk factors for child mental health problems may also relate to parental perceived stigma. More specifically, in line with the definition of the two types of stigma, we expected that parental perceived public stigma would be more susceptible to social–environmental factors than to child or family factors, and that affiliate stigma would primarily relate to parental functioning.

## 2. Materials and Methods

### 2.1. Study Sample and Design

Participants were 317 parents (97% Dutch origin) with a child aged below 18 years referred to one of two child and adolescent psychiatry outpatient clinics with several locations in the Northern or Eastern part of the Netherlands including both rural and urban areas, covering a large catchment area. The current sample concerns the baseline and third wave (about one year later) of a larger three-wave study aimed at collecting data on a large number of child, family, and social–environmental factors that could influence outpatient treatment of children and adolescents referred to the clinic [37]. Children had to be newly referred (i.e., first referral to the respective mental healthcare clinic) to be eligible to participate in the study. Children referred for eating disorders or forensic problems were not invited to the study because treatment trajectories in these settings are not always entirely voluntary. Mental healthcare in the Netherlands is accessible to all families. There were no incentives for a family to follow treatments, which were all voluntary. Data were collected from May 2015 until September 2017. The Supplement S1 describes the data collection procedures and children’s clinical diagnoses in more detail; Figure S1 shows the participation flow of the study. Table 1 presents participants’ characteristics.

### 2.2. Outcome Measures

Parental perceived public stigma and affiliate stigma (self-stigma of the caregiver) were measured by the modified Parents’ Perceived Stigma of Service Seeking scale (PPSSS, [30]; see Supplement S2) during the third wave. The Perceived Public Stigma Scale of the PPSSS refers to perceptions of personal rejection (expected negative treatment from others) due to assumptions about public attitudes of what most people think about mental illnesses or using mental health services. The affiliate stigma scale refers to the ways that parents internalize the public view, resulting in negative beliefs about the self [30]. We adapted the wording of the items to fit the purpose of our study, assessing stigma as perceived by the parents during their child’s treatment trajectory over the past year. We deliberately chose this since the experience of stigma may be influenced by being involved in treatment, as compared to measuring stigma before the start of treatment. We examined the factor structure of the scale (see Supplement S2 and Table S1 for the full principal factor analysis): eight items measured perceived public stigma (observed Cronbach’s α = 0.92; e.g., ‘I was worried some people would treat me with less respect’, ‘I was worried my child might be labelled at school’) as compared to the original 11 items [30]; six items measured affiliate stigma (observed Cronbach’s α = 0.86; e.g., ‘I was embarrassed’, ‘I felt like a bad parent’); all rated on a six-point scale (1 = Strongly disagree; 6 = Strongly agree), a higher score indicated a higher level of perceived public stigma or affiliate stigma.

### 2.3. Predictors

Primary parents’ (i.e., who is most involved in child rearing) answers from baseline were used, unless otherwise stated; treatment-related predictors were answered at the third wave (see Table 1). See Supplementary Table S2 for a detailed description of the scales. All reported Cronbach’s alpha’s were observed in the sample.

#### 2.3.1. Child Characteristics

These included (1) gender; (2) age; (3) general school functioning (Cronbach’s α = 0.73); (4) presence of learning difficulties or mental disability; (5) mental disorder diagnosis during the study course (yes/no); (6) level of callous–unemotional traits using the Inventory of Callous–Unemotional Traits (ICU, [38,39], Cronbach’s α = 0.87); (7) severity of externalizing behavior (Cronbach’s α = 0.78) and (8) of internalizing problems (Cronbach’s α = 0.69) by the Strengths and Difficulties Questionnaire (SDQ, [40]).

#### 2.3.2. Characteristics of the Primary Parent

These included (1) gender, (2) age, (3) physical illness of the primary parent, (4) previous or current mental healthcare use of the primary parent, (5) current mental health state by the self-reported Mental Health Inventory-5 (i.e., depressive and anxiety symptoms, MHI, [41], Cronbach’s α = 0.86), a higher score indicates a better mental health state, and (6) presence of ADHD via the adult ADHD Self-Report Scale (ASRS, [42,43]).

#### 2.3.3. Parenting and Family Characteristics

These included (1) level of involvement in parenting (Cronbach’s α = 0.73) and (2) degree of corporal punishment (Cronbach’s α = 0.53) by the Alabama Parenting Questionnaire (APQ, [44,45,46]); (3) perceived parenting competence by the Parenting Sense of Competence Scale (PSOC, [47], Cronbach’s α = 0.82); (4) parental stress by the Parental Stress Scale (PSS, [48], Cronbach’s α = 0.86); (5) family functioning by the Dutch Parental Questionnaire of Family Functioning (VGFO, [49], Cronbach’s α = 0.90); (6) treatment expectancy of the primary parent (Cronbach’s α = 0.86) and (7) belief in treatment of the primary parent based on the Credibility and Expectancy Questionnaire (CEQ, [50], Cronbach’s α = 0.68); and (8) pretreatment motivation of the primary parent by the Parent Motivation Inventory (PMI, [51], Cronbach’s α = 0.93).

#### 2.3.4. Treatment-Related Characteristics

These included parent-rated assessments at baseline regarding (1) previous mental healthcare use of the child; (2) current non-psychotropic/psychotropic medication use at baseline, open-ended answers were dichotomized; (3) the source of referral: (i) parent(s) or child, (ii) school, or (iii) a health professional (e.g., hospital consultant, general practitioner); and at the third wave regarding (4) type of treatment received in the past year: (i) no treatment, (ii) medication, (iii) behavioral treatment, or (iv) combined medical and behavioral treatment; (5) number of appointments; and (6) behavioral improvement of the child since the start of treatment (SDQ; [40]).

#### 2.3.5. Sociodemographic Characteristics

These comprised (1) single parent household; (2) other children in the household; (3) parent-rated financial problems in the household: (i) present, (ii) absent, or (iii) refusal to answer the question; (4) socio-economic status (SES) derived from five standardized predictors, in accordance with Ganzeboom et al. [52]; (5) urbanicity; and (6) high risk behavior families, calculated by the mean of 22 standardized variables (e.g., drug use of primary parent and/or partner, child came into contact with judicial system); a higher score indicates more deviant behavior of the family.

#### 2.3.6. Social-Environmental Characteristics

Six predictors measured the social embedding of the primary parent: (1) volunteer work; (2) contact via social media; (3) playing sports with others; (4) religious denomination; and (5) contact with neighbors; all dichotomized. Furthermore, we used (6) the intergenerational closure subscale from Sampson and Graif [53] on neighborhood social capital (e.g., parents know each other and look out for each other’s children, Cronbach’s α = 0.76). Lastly, we included (7) deviant behavior and neglect in the neighborhood (e.g., use of drugs on the street, noise nuisance in the neighborhood, Cronbach’s α = 0.92).

### 2.4. Statistical Analyses

In total there were 20 missing cases in two items: urbanicity (3.47% missing) and number of treatment appointments (2.84% missing). These were imputed with five imputations by applying predictive mean matching, using the *mice*-package in R [54,55]. Furthermore, 5 outliers were deleted based on the Mahalanobis distance (1936). Thus, a total of 312 cases were analyzed.

We used logistic least absolute shrinkage and selection operator, i.e., LASSO regression analyses [36] to investigate which variables were associated with respectively perceived public stigma and affiliate stigma in the primary parent. This method is suitable for selecting relevant factors from a large set of variables by automatically assigning a penalized term to standardized variables with a low error. The best fitting penalty term is selected by calculating the average mean cross-validated error (through 10,000 iterations [56]). LASSO shows a good prediction accuracy, reduces overfitting, and mitigates the issue of multicollinearity, and is therefore a sensitive method to investigate a large number of predictors. Analyses were performed using the *glmnet*-package in R [55,57]. All assumptions were met.

To assess the best fitting factors of respectively perceived public stigma and affiliate stigma, two models were used including all predictor variables. Statistical relevance of the predictors was based on non-zero coefficients (i.e., an explained deviance and beta greater than zero). Furthermore, explained deviance of the predictors was based on the leave-one-out method. This method compares the entire model (all predictors) with the entire model minus the predictor of interest. The difference in explained deviance is the contribution to the explained deviance by the predictor of interest [58]. We focused on those predictors with an explained deviance >1%. Notably, due to the shrinkage process of LASSO (i.e., only selecting the best fitting predictors and setting all other predictors to zero), the non-zero beta coefficients are estimates; hence, they should be interpreted with caution since LASSO creates an upward bias for non-zero coefficients.

## 3. Results

### 3.1. Sample Descriptives

As can be seen in Table 1, about two-thirds of the participating children and adolescents were boys. Children’s mean age was around 10 years. The majority of the primary parents were women, with a mean age of around 40 years. About two-thirds of the parents and their child had started treatment (medication, behavioral treatment, or a combination of both), with a mean number of around 10 appointments received in the first year since baseline. Nine out of ten children met the clinical SDQ cut-off for externalizing and/or internalizing mental health problems at baseline; based on the four-band categorization of the severity of externalizing and internalizing mental health problems (i.e., high and very high; https://sdqinfo.org/ (accessed on 25 September 2022)). The average level of perceived public stigma and affiliate stigma was low.

**Table 1:** Descriptive statistics of the study variables answered by the primary parent (*n* = 312).

		Mean/*n*	SD/%	Range
*Outcome variables*				
Perceived public stigma by the primary parent (PPSSS) ^a^		1.77	0.91	1−6
Affiliate stigma in the primary parent (PPSSS) ^a^		1.73	0.91	1−6
*Child characteristics*				
Female gender		109	34.9%	
Age		9.50	3.62	2−17
General school functioning		3.98	0.71	1−5
Presence of learning difficulties		128	41.0%	
Presence of mental illness diagnosis of the child during the course of the study ^a^		230	73.7%	
Level of callous and unemotional traits (ICU)		2.24	0.43	1−4
Severity of externalizing problem behavior (SDQ)		1.93	0.40	1−3
Severity of internalizing problems (SDQ)		1.79	0.39	1−3
Clinical cut-off externalizing and internalizing problems (SDQ) ^b^	No severe mental health problems	26	8.33%	
	Externalizing problems	110	35.3%	
	Internalizing problems	33	10.6%	
	Mixed (both externalizing and internalizing)	143	45.8%	
*Characteristics of the primary parent*				
Female gender		274	87.8%	
Age		40.6	6.50	25−65
Physical illness		84	26.9%	
Previous or current mental healthcare use		150	48.1%	
Mental health (MHI)		4.68	0.83	1–6
Presence of ADHD (ASRS)		26	8.33%	
*Parenting and family characteristics*				
Level of involved parenting (APQ)		3.88	0.45	1−5
Degree of corporal punishment (APQ)		1.57	0.46	1−5
Perceived parenting competence (PSOC)		4.32	0.65	1−6
Parental stress (PSS)		2.02	0.49	1−5
Family functioning (VGFO)		3.27	0.39	1−6
Treatment expectancy of the primary parent (CEQ)		3.83	0.69	1−5
Belief in usefulness of treatment of the primary parent (CEQ)		4.10	0.68	1−5
Pretreatment motivation of the primary parent (PMI)		3.83	0.49	1−5
*Treatment-related characteristics*				
Previous mental healthcare use of child		123	39.4%	
Current medication use at baseline (non-/psychotropic)		56	17.9%	
Source of referral	Parents or child	192	61.5%	
	School	55	17.6%	
	Professional	65	20.8%	
Type of treatment received by child or parents during the course of the study ^a^	None	105	33.7%	
	Behavioral	114	36.5%	
	Medication	53	17.0%	
	Combination of behavioral and medication	40	12.8%	
Number of appointments ^a^		10.3	12.8	0−60
Behavioral improvement of the child ^a^		3.99	0.97	1−5
		**Mean/*n***	**SD/%**	**Range**
*Sociodemographic characteristics*				
Single parent household		44	14.4%	
Other children in the household		267	85.6%	
Financial problems	No	68	21.8%	
	Yes	216	69.2%	
	No answer	28	8.97%	
Socio-economic status		−0.36	0.54	−2.25−1.23
Socio-economic status ^c^	Low	37	11.9%	
	Medium	229	73.4%	
	High	46	14.7%	
Urbanicity		63,869	48,915	7500−195,418
Urbanicity categorized in three categories	Small-sized city (<40,000 inhabitants)	152	48.7%	
	Middle-sized city (40,000–100,000 inhabitants)	90	28.8%	
	Large-sized city (>100,000 inhabitants)	70	22.4%	
High risk behavior families ^c^	Low risk	22	7.05%	
	Normal risk	244	78.2%	
	High risk	46	14.7%	
*Social–environmental characteristics*				
Contact with neighbors		256	82.1%	
Contact via social media		243	77.9%	
Playing sports with others		110	35.3%	
Volunteer work		68	21.8%	
Religious denomination		199	63.8%	
Intergenerational closure in the neighborhood (SCN)		3.78	0.84	1−6
Deviant behavior and neglect in neighborhood		2.00	1.16	1−6

^a^ Measured at the third wave (after one year). Stigma was retrospectively assessed as perceived during the past year while being in care at the outpatient clinic. All other measures assessed at baseline. ^b^ Represents the proportion of children in the clinical range with high or very high scores based on the SDQ (https://sdqinfo.org (accessed on 25 September 2022)). ^c^ Data from primary parent (i.e., who has most parenting time with the child) and secondary parent. Abbreviations: PPSSS = adapted version of Parents’ Perceived Stigma of Service Seeking [30]; ICU = Inventory of Callous–Unemotional Traits [38,39]; SDQ = Strengths and Difficulties Questionnaire [40]; MHI = Mental Health Inventory-5 [41]; ASRS = ADHD Self-Report Scale [42,43]; APQ = Alabama Parenting Questionnaire [44,45,46]; PSOC = Parental Sense of Competence Scale [47]; PSS = Parental Stress Scale [48]; VGFO = Parental Questionnaire Family Functioning [49]; CEQ = Credibility and Expectancy Questionnaire [50]; PMI = Parental Motivation Inventory [51]; SCN = Social Capital in the Neighborhood [53].

### 3.2. LASSO Regression

Table 2 shows the LASSO regression prediction model with the corresponding explained deviances and betas of all relevant factors (i.e., explained deviance >1%; see Supplementary Table S3 for the factors below 1%). In total, eight out of 45 factors were relevant in the model regarding parental perceived public stigma and one factor regarding affiliate stigma. Each of these individual factors had a small effect. We found a total explained deviance for perceived public stigma of 34.0% and for affiliate stigma of 19.7% for all individual factors combined. Out of the six predictor domains, child and social–environmental characteristics explained the most deviance regarding perceived public stigma, followed by parental factors. The only relevant factor of affiliate stigma was found for parenting characteristics. We found no relevant factors in the sociodemographic and treatment-related domains.

### 3.3. Child Characteristics

Child characteristics explained 5.0% of the total deviance in perceived public stigma and none in affiliate stigma in the parents. The more severe the child’s problems, the higher the perceived public stigma; that is, the level of callous–unemotional traits and severity of internalizing problems had a relatively large effect in the model. Moreover, we found that having an older child was related to a lower level of parental perceived public stigma.

### 3.4. Characteristics of the Primary Parent

The total explained deviance of characteristics of the primary parent was 2.2% for perceived public stigma and 1.2% for affiliate stigma. The only relevant effect was previous or current use of mental healthcare of the primary parent associated with higher perceived public stigma.

### 3.5. Parenting and Family Characteristics

The total explained deviance of parenting and family characteristics for perceived public stigma was 1.8% and for affiliate stigma 2.4%. Perceived parenting competence was the strongest predictor in both respective models; a higher perceived parenting competence was related to a lower level of perceived public and affiliate stigma. All other parenting and family factors were not relevant in either model.

### 3.6. Treatment-Related Characteristics

Treatment-related characteristics explained 1.2% of the deviance of perceived public stigma and 0% of affiliate stigma. There were no relevant treatment-related characteristics related to stigma.

### 3.7. Sociodemographic Characteristics

Sociodemographic characteristics explained 2.1% of deviance in perceived public stigma and 0.3% in affiliate stigma. However, no single sociodemographic characteristic appeared relevant above the 1% threshold.

### 3.8. Social-Environmental Characteristics

A total of 3.9% of deviance was explained in the perceived public stigma model by social–environmental characteristics. Deviant behavior and neglect in the neighborhood (e.g., use of drugs on the street, noise nuisance in the neighborhood) was related to a higher level of perceived public stigma. In contrast, belonging to a religious denomination and a higher intergenerational closure (e.g., parents know each other and look out for each other’s children) predicted lower perceived public stigma.

## 4. Discussion

Stigma is of major concern in child mental healthcare as it may affect the child’s treatment process [10,15,16]. While parents play an important role in their child’s treatment, factors that contribute to parental perceived stigma are understudied, and so far it has been unclear which factors may be the most important ones. To provide a synthesis of previously indicated factor domains, we investigated a broad set of child, family, and social–environmental factors in relation to perceived public stigma (i.e., parents’ perceptions of stereotypical beliefs held by the larger public) and affiliate stigma (i.e., internalization of stereotypical beliefs by the parent applying these to him/herself) [7] of primary caregivers of children and adolescents relating to the first year after their child’s referral to an outpatient mental health clinic.

Most effects were found for factors in relation to parents’ perceived public stigma, although the contribution of each individual factor was small. The most important factors for *higher* perceived public stigma were: (1) child’s problems, (2) previous or current mental healthcare use of the parent, and (3) deviant behavior and neglect in the neighborhood. Furthermore, the most important factors for a lower perceived public stigma were: (1) higher perceived parenting competence of the parent, (2) older age of the child, and (3) more social support (i.e., religious denomination and intergenerational closure in the neighborhood). Only one relevant factor related to (a lower) affiliate stigma: perceived parenting competence of the parent.

In line with previous studies, parents perceived more public stigma when their child had a higher problem severity [18,31,59]. Interestingly, in our study, higher callous–unemotional traits (often linked to “child psychopathy” or conduct disorder) came forth as the most important factor rather than the broader dimension of externalizing problems, underscoring the importance of this trait in children with disruptive problems [60]. Similarly, also in children with disabilities, parents’ perceived stigma was related to their child’s lower level of prosocial behaviors [59]. A possible explanation for the higher perceived public stigma is that parents may have the feeling that their child’s behavior problems are not considered to be a serious mental health problem by others [61], perhaps blaming the parents for their child’s behavior [14]. In the ‘National Stigma Study-Children’, externalizing problem behavior (ADHD) was considered less of a serious mental illness by the general public, compared to internalizing problems such as depression [61], although the severity of internalizing problems was also related to perceived public stigma in our study (see also [1,19,59], albeit less strongly). Additionally, Calear et al. [62] found that some types of anxiety disorders (e.g., social phobia) in adolescents were more frequently viewed as a sign of personal weakness, rather than a ‘real medical illness’ such as depression; highlighting that increased public stigma can be associated with some anxiety disorders.

The finding that the previous use of mental healthcare of the parent was related to a higher perceived public stigma is not in line with the adult literature, which showed that the previous use of mental healthcare is related to both a lower level of stigmatizing attitudes and a greater willingness to seek professional help [63,64]. However, in our study, parent’s experiences with mental healthcare regarding their own problems may have made them more aware of negative attitudes towards mental problems or healthcare use of their child held by the general public, increasing feelings of stigmatization concerning their child. Moreover, the higher perceived stigma in parents who have received mental healthcare for their own problems is consistent with previous studies that linked it to parent’s lower wellbeing [29,32], although we did not find a relation of perceived stigma with parent’s current anxiety and depressive symptoms [19,20].

It may not be surprising that deviant behavior and neglect in the neighborhood (e.g., use of drugs, noise in the street) were also related to a higher perceived public stigma in parents; previous research has shown that people with a low socio-economic status, who more often live in disadvantaged areas, hold more stigmatizing attitudes towards a mental illness [63,65,66]. This may in turn affect parents’ perception of public stigma regarding their child. It has also been shown that individuals with psychiatric disorders living in a neighborhood with strong mutual support reported lower perceived stigma, whereas a neighborhood with a large number of psychiatric patients with disabilities reported higher levels of perceived public stigma (see [67]).

Consistent with the above, a higher level of social support, as reflected by belonging to a religious denomination and a better intergenerational closure in the neighborhood (i.e., parents know each other and look out for each other’s children), was related to a lower perceived public stigma in the parent. This is in line with previous studies [19,32,35,67] demonstrating lower stigma if a person has more social interaction (e.g., family, friends, neighborhood, or communities). For example, Chang et al. [25] reported a trend for lower affiliate stigma associated with a higher frequency of attending religious activities. In turn, it has been shown that more positive attitudes are formed towards stigmatized groups upon increased contact with them, supporting social cohesion [8,64]. A ‘tight-knit’ social network might disprove the belief of a parent that the general public holds stereotypical views of his or her child, and mitigate feelings of isolation and rejection [2], thus protecting against the perception of stigma.

The finding that a higher parenting sense of competence of the parent was associated with a lower perceived public and affiliate stigma is in line with previous findings, suggesting a protective effect [6,59]. Perhaps, parents who perceive themselves as competent parents feel less stigma because they do not see themselves as ‘bad parents’, having a higher level of psychological health and a more positive self-evaluation [68,69], making them less vulnerable to others’ judgements. While in one study, specifically lower bad parent beliefs (a core aspect of affiliate stigma) were related to parents’ empowerment [70], our study indicates that a positive self-attitude as a parent expands to a lower susceptibility to public stigma. In a study of children with disabilities, the authors suggested that helping parents to deal with public stigma and increase their parenting self-efficacy will support the child’s development [59].

Finally, parents of older children also perceived less stigma, consistent with an earlier finding [31]. As an explanation, it was suggested that older parents (who tend to have older children) may become less sensitive to stigmatization over time [31]; however, we could not confirm that parents’ older age was related to less perceived stigma. Our finding may be better understood by the greater stigmatization of parents with younger children with mental health problems [61]; this has been explained by younger parents holding more stigmatizing attitudes rather than the child’s age being the target of stigmatization [28]. Thus, contact with the peer group might explain why parents of younger children are more likely to perceive public stigma.

In contrast to our expectations, we did not find relations of most parent- or family-related factors with affiliate stigma. Previous studies indicated that parents’ affiliate stigma was related to a range of parental factors, such as self-esteem, parental stress, or family cohesion [18,33,34]. This might point to a comparatively better psychological health of our study participants, perhaps reflecting selection bias with the inclusion of more well-functioning families. It may also be possible that affiliate stigma may gain greater importance as time passes from when a diagnosis was given [33], considering that our sample included newly referred families and given that affiliate stigma largely develops from the internalization of public stigma over time [71]. Finally, sociodemographic and treatment-related characteristics were not related to parents’ perceived public and affiliate stigma in our study; this is in line with earlier findings [72], although in one study caregivers of children with ADHD with a higher education level reported higher affiliate stigma [25].

### 4.1. Strengths and Weaknesses

A strength of this study was the large sample of families referred to a child and adolescent psychiatry outpatient clinic using a sophisticated quantitative method to select the most relevant factors associated with parents’ perceived public stigma and affiliate stigma. While previous research mainly focused on affiliate stigma and specific childhood mental disorders, this study investigated which factors relate to perceived public stigma and affiliate stigma in parents in a cross-section of referred families in outpatient child and adolescent mental healthcare, including a wide range of youth mental health problems. Therefore, we were able to address the heterogeneity of perceived public stigma and affiliate stigma in parents of a child with emotional and/or behavioral problems.

Our study also has some limitations, among which is the generalizability of findings considering likely sample differences. Furthermore, ratings of predictor and outcome measures were both from the primary parent and thus not independent. Moreover, as we did not measure stigma at study entry and the change in stigma over time, but retrospectively reflected on perceived stigma during the first year after referral, we cannot exclude recall bias (e.g., parents may have indicated less perceived stigma after one year since referral than they actually perceived during the past year), which may perhaps explain the relatively low level of perceived stigma in our study (low levels were also previously reported in a community sample [30]). Causal inferences cannot be made due to the cross-sectional data. Furthermore, although we found a similar factor structure of public and affiliate stigma in our clinic-referred mixed rural–urban sample using the PPSSS [30], future studies should further validate the measure including the measurement invariance. In addition, LASSO regression has the limitation that it randomly selects one predictor if factors are correlated [73]. We minimized this by running the model 10,000 times to select the best error term in a similar way to the stability selection technique described by Meinshausen and Bühlmann [56]. Lastly, we did not assess the child’s self-stigma, another important understudied aspect of stigma.

### 4.2. Conclusions

In conclusion, our study confirms that a multitude of factors are related to perceived parental stigma as has previously been acknowledged [25]. Notably, a child’s higher level of callous–unemotional traits appeared to evoke more perceived public stigma in parents, as did more severe internalizing symptoms. Additionally, a history of mental healthcare use of the parent played a role, as well as social–environmental factors, namely social relations and neighborhood neglect. Both perceived public stigma and affiliate stigma were associated with parenting sense of competence. Clinicians should be aware of potential perceived stigma in parents and help them cope to feel less stigmatized. Clinicians may therefore seek to identify the set of factors relevant to the particular parents. In addition, by strengthening parents’ parenting competence, for example, in parent training programs, perceived stigma might be reduced, which should be further explored in future studies. Other important steps for future research will be to investigate how parents’ perceived stigma may influence their response to their child’s treatment and quality of life.

## Figures and Tables

**Table 2:** Relevant predictors of parents’ perceived public stigma and affiliate stigma (PPSSS, *n* = 312) ^a^.

	Perceived Public Stigma ^b^		Affiliate Stigma ^b^	
	Explained Deviance	β (%)	Explained Deviance	β (%)
*Child characteristics*				
Age	1.17	−0.15	-	0.00
Level of callous–unemotional traits (ICU)	1.85	0.13	-	0.00
Severity of internalizing problems (SDQ)	1.32	0.10	-	0.00
*Characteristics of the primary parent*				
Previous or current mental healthcare use	1.72	0.13	-	0.00
*Parenting and family characteristics*				
Perceived parenting competence (PSOC)	1.54	−0.20	1.00	−0.15
*Social–environmental characteristics*				
Religious denomination	1.02	−0.09	-	0.00
Intergenerational closure in neighborhood (SCN)	1.01	−0.09	-	0.00
Deviant behavior and neglect in neighborhood	1.05	0.07	-	0.00

^a^ Factors that met the cut-off point of 1% in the LASSO regression analyses. See Supplementary Table S3 for the irrelevant factors. All data by the primary parent (i.e., who has most parenting time with the child). ^b^ Measured at the third wave, retrospectively assessing stigma as perceived during the past year while being in care at the outpatient clinic. All other measures assessed at baseline. Abbreviations: PPSSS = adapted version of the Parents’ Perceived Stigma of Service Seeking [30]; ICU = Inventory of Callous–Unemotional Traits [38,39]; SDQ = Strengths and Difficulties Questionnaire [40]; PSOC = Parental Sense of Competence Scale [47]; SCN= Social Capital in the Neighborhood [53].

## Data Availability

Data are not publicly available due to ongoing analyses. The data can be made available upon reasonable request to the corresponding author.

## Supplementary Materials

The following supporting information can be downloaded at: https://www.mdpi.com/article/10.3390/ijerph191912767/s1, Supplement S1. Description of data collection and clinical diagnoses. Supplementary Figure S1. Participation flow of the three-wave study. Supplement S2. Principal factor analysis on the Parent’s Perceived Stigma of Service Seeking (PPSSS) scale. Supplementary Table S1. Pattern matrix and communalities of the 17 items of the modified PPSSS scale. Supplementary Table S2. Description of scales used in this study. Supplementary Table S3. Irrelevant predictors of parents’ perceived public stigma and affiliate stigma. References [30,38,39,40,41,42,43,44,45,46,47,48,49,50,51,52,53] are cited in the supplementary materials.
