# Supplementary material for: Factors Related to Perceived Stigma in Parents of Children and Adolescents in Outpatient Mental Healthcare
Source: Int J Environ Res Public Health. 2022 Oct 6;19(19):12767. doi: 10.3390/ijerph191912767 (PMC9566109; doi:10.3390/ijerph191912767)
Supplement: Supplementary file 1 [file ijerph-19-12767-s001.zip › ijerph-1847256-supplementary.pdf]

Title: Factors Related to Perceived Stigma in Parents of Children and Adolescents in Outpatient Mental Healthcare

Halewijn M. Drent [1,2], Barbara J. van den Hoofdakker [1,2], Jan K. Buitelaar [3], Pieter J. Hoekstra [1,2] and Andrea Dietrich [1,2,*]

[1] Department of Child and Adolescent Psychiatry, University Medical Center Groningen, University of Groningen, 9723 HE Groningen, The Netherlands
[2] Accare Child Study Center, 9723 HE Groningen, The Netherlands
[3] Radboud University Medical Center, Donders Institute for Brain Cognition and Behaviour, Department of Cognitive Neuroscience, 6525 AJ Nijmegen, The Netherlands; j.buitelaar@donders.ru.nl
* Correspondence: a.dietrich@accare.nl

Supplement S1. Description of data collection and sample characteristics.

Families were approached to participate in the study between the beginning of May 2015 and the end of September 2017 by mailed letters together with their first invitation letter to the outpatient child and adolescent mental health clinic. After consenting to participate, we asked the families to fill out the first set of questionnaires before a possible treatment started. This first set of questionnaires included a questionnaire for the primary parent (i.e., the primary caregiver in the household; ~75 min), the secondary parent (i.e., partner of the primary parent in the same household; ~30 min), and the child or adolescent (≥8 years old; ~20 min). The second set of questionnaires was sent about six months after completion of the baseline questionnaires, and only to the primary parent (~25 min) and the child/adolescent (~15 min). About six months later, the third set of questionnaires was sent to all primary parents (~30 min) and children/adolescents (~15 min) who completed the questionnaire. Data were obtained via online questionnaires (paper versions were sent on request). E-mail reminders to complete the questionnaire were sent two and three weeks after the invitation had been sent. Families who then still did not respond were reminded by telephone to participate. A voucher of 20 euros was sent to each family after completion of each wave. Participation in the study was voluntary. See Figure S1 for the participation flow.

Children and adolescents who participated in both the baseline and third wave were on average 0.7 years younger than those who only participated at baseline ($t(432) = -5.22$, $p < 0.001$). In addition, primary parents who participated in both waves were on average 0.6 years younger ($t(519) = -2.48$, $p = 0.014$). Furthermore, more mothers ($t(371) = -2.29$, $p = 0.023$) and boys ($t(420) = -2.36$, $p = 0.019$) participated in the third wave compared to baseline. No significant differences were found for single parent household ($t(519) = 0.63$, $p = 0.527$) and socio-economic status ($t(519) = -0.15$, $p = 0.88$).

The primary parent reported the clinical diagnosis of their child assessed by the mental health care provider from the outpatient clinic. Of the 312 participants in the present study, the following psychiatric diagnoses were given: 38.4% attention-deficit/hyperactivity disorder (ADHD), 24.1% autism spectrum disorder (ASD), 13.9% anxiety disorder, 2.4% mood disorder, 9.5% other diagnoses (e.g. conduct disorder, obsessive-compulsive disorder, tic disorder, mental disability) and 11.6% did not receive a psychiatric diagnosis.

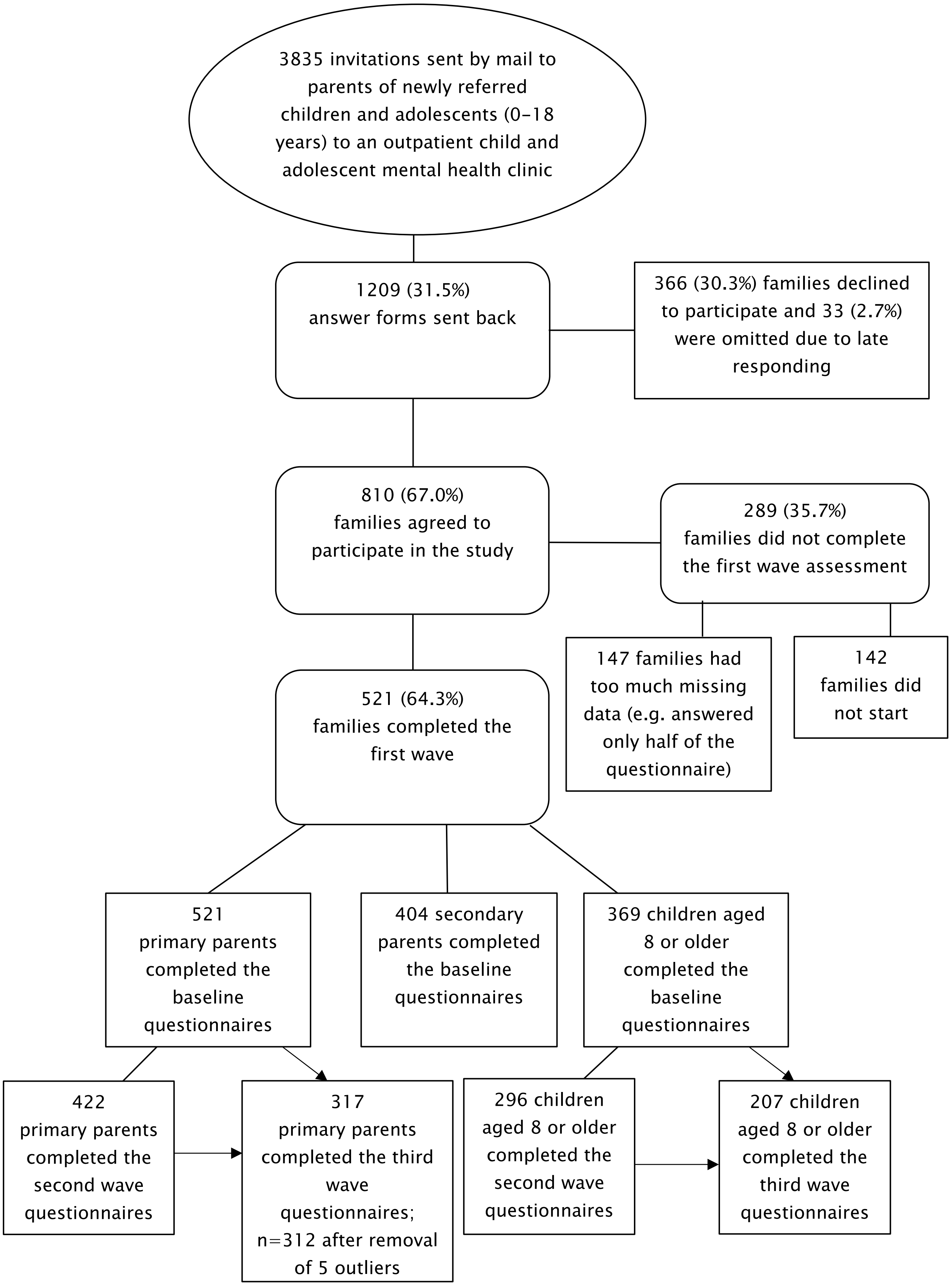

Supplementary Figure S1. Participation flow of the three-wave study on the influence of child, family, and social–environmental factors on response to treatment in outpatient child and adolescent mental healthcare.

Supplement S2. Principal factor analysis on the Parent's Perceived Stigma of Service Seeking (PPSSS) scale.

We examined the factorability of a modified version of the Parent's Perceived Stigma of Service Seeking scale (PPSSS; Williams and Polaha, 2014) [1] using the original number of 18 items by principal factor analysis using oblique rotation. The PPSSS was designed to measure parents' perceptions of stigma about seeking mental health services for their children and was tested in a sample experiencing psychosocial concerns and in a general population sample in a rural area, identifying two factors, parents' perceived public and affiliate (or self) stigma. For the purpose of our study measuring stigma while receiving treatment within the first year after reference to a child and adolescent outpatient clinic, we reworded items to past tense since participants had already entered mental healthcare rather than being at the stage of seeking help; fitting the period in which the stigma took place (for example, we changed the wording from 'It would make me feel strange' to 'I felt strange'; or from 'I would be worried' to 'I was worried'). We also adjusted the original item 14 'People in my church might frown on my decision' to 'I was worried people in my surroundings might frown on my decision' to better reflect a more secular, urban environment.

In line with Williams and Polaha (2014)[1], we removed one item from the principal factor analysis: 'I think there is something wrong with my child' (item 7), due to a communality below 0.30. There were no further violations of the other items (all items had a correlation of 0.30 with at least one other item, anti-image correlation diagonals were all above 0.50, the Kaiser–Meyer–Olkin measure of sampling adequacy was higher than 0.60 (0.92 in our sample), and Bartlett's test of sphericity was significant ($\chi 2(153) = 4674.68$, $p < 0.001$)). We then recalculated the principal factor analysis with the 17 items. No violations were found: (1) all items had a correlation higher than 0.30 with at least one other item, (2) the anti-image correlation diagonals were all above 0.50, (3) the Kaiser–Meyer–Olkin measure of sampling adequacy remained high (0.92), (4) Bartlett's test of sphericity was significant ($\chi 2(136) = 4552.25$, $p < 0.001$)). Furthermore, as shown in Table 1, all communalities were higher than 0.30, and there were no cross loadings.

Initial eigenvalues indicated there were three underlying factors, explaining 52.0%, 10.9%, and 8.97% of the variance, respectively, in contrast with Williams and Polaha (2014)[1] who reported a two factor model. As shown in Table 1, the 11 items intended to measure perceived public stigma reflected two different factors: one factor measuring perceived public stigma (8 items) and one measure that seemed to be conceptualizing the 'willingness to take action' upon perceiving stigma (3 items: items 16, 17, 18). We likely identified a separate factor in our mixed rural–urban sample since these three items may be more relevant in a rural setting. Although the scale perceived public stigma covers perceived personal rejection toward self (parent) and (child), both aspects load on a common factor. Furthermore, in line with Williams and Polaha (2014)[1], we found six items that specify affiliate stigma (called self-stigma by the authors). As shown in Supplementary Table 1, the internal consistency (Cronbach's alpha) for each of the scales was good. Lastly, we found a Pearson correlation of 0.65 between the affiliate stigma and public perceived stigma subscales, a correlation of 0.52 between the affiliate stigma and attitude to action subscales, and a correlation of 0.44 between the perceived public stigma and attitude to action subscales. Notably, in our sample, the items defining affiliate stigma had a negative loading on the pattern matrix, which indicates that if a parent possesses more of the construct 'affiliate stigma', he/she possesses less of the underlying factor of all items combined.

Supplementary Table S1. Pattern matrix and communalities of the 17 items of the modified PPSSS used in parents of referred children and adolescents to an outpatient mental health clinic after 1 year of referral (*n* = 317).

Items	*Perceived public stigma*	*Attitude to action*	*Affiliate stigma*	*Communalities*
1. I felt strange			−0.63	0.53
2. I was embarrassed			−0.75	0.69
3. I felt like a bad parent			−0.81	0.69
4. My view of myself was less			−0.72	0.67
5. It felt that I am weak			−0.84	0.76
6. It felt like there was something wrong with me			−0.82	0.69
7. I think there is something wrong with my child	-	-	-	<0.30
8. I was worried some people might treat me unfairly	0.70			0.69
9. I was worried some people might look down on me	0.75			0.80
10. I was worried some people might say bad things about me behind my back	0.90			0.86
11. I was worried some people would treat me with less respect	0.89			0.87
12. I was worried some people would avoid me	0.84			0.74
13. I was worried my child might be labelled at school	0.52			0.29
14. I was worried people in my surroundings might frown on my decision	0.68			0.55
15. I was worried my child's teacher would treat him or her unfairly	0.57			0.33
16. I was worried that people in town would find out		0.83		0.84
17. I tried to hide that I was getting counseling for my child		0.86		0.80
18. I tried to go to a counselor in another town so no one I know would find out		0.62		0.47
Cronbach's alpha	0.92	0.91	0.86	

PPSSS = Parent's Perceived Stigma of Service Seeking scale (Williams and Polaha, 2014).

Supplementary Table S2. Description of scales used in this study.

Measure	*Number of items (range of scale items)*	*Scale description and item samples*	*Cronbach's α in validated study (Cronbach's α in this study)*	*Notes*
Outcome measures				
Parents' Perceived Stigma of Service Seeking (PPSSS)[1]	18 (1 = Strongly disagree; 6 = Strongly agree	Addresses perceived public stigma and self-stigma of parents seeking help for their child. Perceived public stigma is based on 12 items (e.g., 'I was worried some people might treat me unfairly', 'I was worried my child's teacher would treat him or her unfairly') and self-stigma was based on 6 items (e.g., 'I felt strange', 'I felt like a bad parent').	Perceived public stigma: (0.92); Self-stigma/Affiliate stigma: (0.86)	We used an adapted version of the PPSSS; see Supplement 1 for the scale construction.
Child characteristics				
General functioning at school	5 (1 = Not at all true; 5 = Entirely true)	Self-constructed scale. Measures behavioral functioning at school. Based on five questions: (1) 'Tries his/her best at school'; (2) 'Never skips a class'; (3) 'Always does his/her homework'; (4) 'Stays home often due to illness', and (5) 'Enjoys going to school'.	(0.73)	
Inventory of Callous–Unemotional Traits (ICU)[2,3]	24 (1 = Not at all true; 4 = Definitely true)	Measures callous, uncaring, and unemotional traits, for example, 'Expresses his/her feelings openly'; 'Does not care about being on time'; 'Always tries his/her best'.	0.83 (0.87)	
Strength and Difficulties Questionnaire (SDQ): internalizing and externalizing subscale[4]	25 (1 = Not true; 3 = Certainly true).	Addresses emotional and behavioral symptoms in the past six months. Item examples are 'Considerate of other people's feelings'; 'Often has temper tantrums or hot tempers'; 'Easily distracted, concentration wanders'; and 'Often lies or cheats'.	Internalizing: 0.84–0.87 (0.68) Externalizing: 0.87–0.93 (0.78)	The internalizing scale consists of 10 items (mean of the 'emotional problems' and 'peer problems' subscales). The externalizing scale consists of 10 items (mean of the 'Conduct problems' and 'Hyperactivity' subscales).

Supplementary Table S2. Description of scales used in this study—continued.

Measure	*Number of items (range of scale items)*	*Scale description and item samples*	*Cronbach's α in validated study (Cronbach's α in this study)*	*Notes*
Characteristics of the primary parent				
Mental Health Inventory-5[5]	5 (1 = Never; 6 = Always)	Addresses parental mental health in the past month; depressive symptoms, anxiety, burn-out. Item examples: 'How much of the time, during the last month, have you been a very nervous person?'; 'How much of the time, during the last month, have you felt calm and peaceful?'; 'How much of the time, during the last month, have you been a happy person?'	0.79–0.88 (0.86)	
Adult ADHD self-report scale ASRS[6,7]	6 (1 = Never; 5 = Always)	Measures the presence of ADHD in parents via six items: (1) 'How often do you have trouble wrapping up the final details of a project, once the challenging parts have been done?'; (2) 'How often do you have difficulty getting things in order when you have to do a task that requires organization?'; (3) 'How often do you have problems remembering appointments or obligations?'; (4) 'When you have a task that requires a lot of thought, how often do you avoid or delay getting started?'; (5) 'How often do you fidget or squirm with your hands or feet when you have to sit down for a long time?'; and (6) 'How often do you feel overly active and compelled to do things, like you were driven by a motor?'		Based on the symptom severity, the six items were classified as the specific symptom being absent (0) or present (1). If primary parents had a summed score of at least 4, they were classified as ADHD.

Supplementary Table S2. Description of scales used in this study—continued.

Measure	*Number of items (range of scale items)*	*Scale description and item samples*	*Cronbach's α in validated study (Cronbach's α in this study)*	*Notes*
Parenting and family characteristics				
Alabama Parenting Questionnaire (APQ): level of involved parenting and corporal punishment subscales [8,9,10]	42 (1 = Never; 5 = Always)	Measures five dimensions of parenting, namely (1) involved parenting, (2) positive parenting, (3) poor monitoring, (4) inconsistent disciplining, and (5) corporal punishment. Example items: 'You have a friendly talk with your child.'; 'Your child fails to leave a note or to let you know where he/she is going.'; 'You feel that getting your child to obey you is more trouble than it's worth.'	Positive parenting: 0.79–0.82<br>Parental involvement: 0.74–0.81<br>Poor monitoring/supervision: 0.81–0.83<br>Corporal punishment: 0.79–0.83<br>Inconsistent disciplining: 0.54–0.62	The level of involved parenting subscale consists of ten items (e.g., 'You have a friendly talk with your child'; 'You talk to your child about his/her friends') and the corporal punishment subscale consists of three items (e.g., 'You spank your child with your hand when he/she has done something wrong').
Parenting Sense of Competence Scale (PSOC)[11]	16 (1 = Strongly disagree; 6 = Strongly agree)	Measures parenting competence. Example items are 'My mother/father was better prepared to be a good mother/father than I am.'; 'Being a parent is manageable, and any problems are easily solved.'; 'Sometimes I feel like I'm not getting anything done'	0.58–0.82 (0.82)	
Parental Stress Scale (PSS)[12]	18 (1 = Strongly disagree; 5 = Strongly agree)	Assesses stress with parenting. Example items: 'I am happy in my role as a parent'; 'Having children has been a financial burden'; 'I feel overwhelmed by the responsibility of being a parent'.	0.83 (0.85)	
Parental Questionnaire Family Functioning (VGFO)[13]	30 (1 = Not true; 4 = Entirely true)	Addresses five dimensions of family functioning, namely (1) basic care, (2) nurturing, (3) social contacts, (4) own youth experiences, and (5) relationship with partner. Example items: 'I give my children enough freedom'; 'I have had a great childhood'; 'my parents had too little time for me'	0.90 (0.89)	

Supplementary Table S2. Description of scales used in this study—continued.

Measure	*Number of items (range of scale items)*	*Scale description and item samples*	*Cronbach's α in validated study (Cronbach's α in this study)*	*Notes*
Credibility and Expectancy Questionnaire (CEQ)—Expectancy of treatment[14]	2 (1 = Completely disagree; 5 = Completely agree)	Two items measured expectancy of treatment: 'At this point, how successful do you think the treatment will be in reducing your child's problems?' and 'I believe my child's problem behavior will have improved at the end of the treatment'.	(0.86)	
Credibility and Expectancy Questionnaire [CEQ]—Belief in treatment[14]	2 (1 = Completely disagree; 5 = Completely agree)	Two items measured belief in treatment: 'For me, treatment acts as a barrier rather than a solution' and 'I think I and my environment are better suited to take care of my child's problems than a therapist'.	(0.68)	
Parental Pretreatment Motivation Inventory (PMI)[15]	25 items (1 = Strongly disagree; 5 = Strongly agree)	Addressing the need to change (e.g. 'My child's behaviour has to improve soon'), readiness to change ('I am willing to work on changing my own behaviour as it relates to managing my child'), and perceived ability to change ('I believe that my child's behaviour cannot change without my involvement in treatment')	0.86 (0.93)	One item was removed from the scale (i.e. 'I am motivated to change the way I reward and punish my child if it will lead to improvement') because there were too many missing responses on this item.
Sociodemographic characteristics				
Socio-economic status (SES)[16]	5 items	Socio-economic status assessed through a standardized composite score of the net income level of the household and the educational and occupational level of both parents.		
High risk behavior Families	Yes/no	Do you own a car?		Categorisation based on self-constructed scale. High risk behavior families is calculated by the average mean of the 22 standardized variables.
	Yes/no	Sufficient monthly net income		
	Yes/no	Financial troubles now		
	Yes/no	Financial troubles in the past		
	0–40 glasses per week	Alcohol use primary parent		

Supplementary Table S2. Description of scales used in this study—continued.

Measure	*Number of items (range of scale items)*	*Scale description and item samples*	*Cronbach's α in validated study (Cronbach's α in this study)*	*Notes*
	0–40 glasses per week	Alcohol use secondary parent		
	Yes/no	Drug use primary parent now		
	Yes/no	Drug use primary parent in the past		
	Yes/no	Drug use secondary parent now		
	Yes/no	Drug use secondary parent in the past		
	Yes/no	Child came into contact with judicial system (last six months)		
	Yes/no	Child came into contact with judicial system (in the past)		
High risk behavior Families	Yes/no	Do you own a car?		Categorisation based on self-constructed scale. High risk behavior families is calculated by the average mean of the 22 standardized variables.
	Yes/no	Sufficient monthly net income		
	Yes/no	Financial troubles now		
	Yes/no	Financial troubles in the past		
	0–40 glasses per week	Alcohol use primary parent		
	0–40 glasses per week	Alcohol use secondary parent		
	Yes/no	Drug use primary parent now		
	Yes/no	Drug use primary parent in the past		
	Yes/no	Drug use secondary parent now		
	Yes/no	Drug use secondary parent in the past		
	Yes/no	Child came into contact with judicial system (last six months)		
	Yes/no	Child came into contact with judicial system (in the past)		
	Yes/no	Lawsuit (past six months)		
	Yes/no	Lawsuit (in the past)		
	Yes/no	Court custody of the child (in the past)		

Supplementary Table S2. Description of scales used in this study—continued.

Measure	***Number of items (range of scale items)***	***Scale description and item samples***	***Cronbach's α in validated study (Cronbach's α in this study)***	***Notes***
	Yes/no	Came into contact with judicial system (in the past six months)		
	Yes/no	Ever arrested (past six months)		
	Yes/no	Ever arrested (in the past)		
	Yes/no	Criminal record primary parent		
	Yes/no	Criminal record secondary parent		
	Yes/no	Psychopathology primary parent		
	Yes/no	Psychopathology secondary parent		
Social–environmental characteristics				
Social Capital in Neighborhood (SCN)—Intergenerational closure in neighborhood[17]	5 (1 = Completely disagree; 5 = Completely agree)	The intergenerational closure subscale measured the social control parents have over each other's children. Item samples: 'Other parents looking out over children in the neighborhood', 'Parents in this neighborhood generally know each other'.	(0.87)	
Deviant behavior and neglect in neighborhood	7 (1 = Completely disagree; 5 = Completely agree)	Self-constructed scale. We constructed a scale of seven items reflecting deviant behavior and neglect in the neighborhood. The seven items were: (1) 'There is often a fight in our neighborhood'; (2) 'There is a lot of disturbance because of loitering in our neighborhood'; (3) 'In our neighborhood, people drink alcohol on the street'; (4) 'Houses could be better maintained'; (5) 'There should be more playing fields/equipment for children'; (6) 'There is a lot of noise disturbance in our neighborhood'; (7) 'In our neighborhood, people do drugs on the street'.	(0.92)	

Supplementary Table S3. Irrelevant predictors of parents' perceived public stigma and affiliate stigma (PPSSS, *n* = 312) [a].

	Perceived public stigma [b]		*Affiliate stigma* [b]	
	Explained deviance (%)	*β*	*Explained deviance (%)*	*β*
Child characteristics				
Female gender	-	0.00	-	0.00
General school functioning	0.28	−0.03	-	0.00
Presence of learning difficulties	-	0.00	-	0.00
Presence of mental illness diagnosis of the child during the course of the study[b]	0.42	0.05	-	0.00
Severity of externalizing problem behavior (SDQ)	-	0.00	-	0.00
Characteristics of the primary parent				
Female gender	0.22	0.03	-	0.00
Age	0.26	−0.07	-	0.00
Physical illness	0.04	0.02	-	0.00
Mental health (MHI)	-	0.00	0.54	−0.08
Presence of ADHD (ASRS)	-	0.00	0.62	0.04
Parenting and family characteristics				
Level of involved parenting (APQ)	-	0.00	-	0.00
Degree of corporal punishment (APQ)	0.10	0.02	-	0.00
Parental stress (PSS)	-	0.00	0.52	0.09
Family Functioning (VGFO)	0.13	−0.03	0.85	−0.07
Pretreatment expectancy of the treatment of the primary parent (CEQ)	-	0.00	-	0.00
Pretreatment belief in the usefulness of treatment of the primary parent (CEQ)	0.03	−0.009	-	0.00
Pretreatment motivation of the primary parent (PMI)	-	0.00	-	0.00
Treatment-related characteristics				
Previous mental healthcare use of child	0.75	−0.06	-	0.00
Current medication use of child (non-/psychotropic)	0.02	0.003	-	0.00
Source of referral (ref. = parents or child)				
School	0.15	0.02	-	0.00
Professional	-	0.00	-	0.00
Type of treatment received during the course of the study (ref. = none)[b]				
Behavioral	-	0.00	0.02	0.0009
Medication	-	0.00	-	0.00
Combination of behavioral and medication treatment	0.23	−0.02	-	0.00
Number of appointments during the course of the study[b]	0.01	0.005	-	0.00
Behavioral improvement of the child one year after referral[b]	-	0.00	-	0.00
Sociodemographic characteristics				
Single parent household	0.25	−0.04	-	0.00
Other children in the household	-	0.00	-	0.00
Financial problems (ref. = no)				
Yes	0.28	−0.04	-	0.00
No answer	0.35	−0.03	-	0.00
Socio-economic status[c]	0.03	0.003	0.32	−0.03
Urbanicity (ref.= small-sized city (< 40,000 inhabitants))	0.75	−0.06	-	0.00
High risk behavior families[c]	0.48	0.05	-	0.00

Supplementary Table S3. Irrelevant predictors of parents' perceived public stigma and affiliate stigma—continued.

	Perceived public stigma[b]		*Affiliate stigma*[b]	
	Explained deviance (%)	*β*	*Explained deviance (%)*	*β*
Social–environmental characteristics				
Contact with neighbors	0.77	0.06	-	0.00
Contact via social media	-	0.00	-	0.00
Playing sports with others	-	0.00	-	0.00
Volunteer work	-	0.00	-	0.00

[a] Factors that did not meet the cut-off point of 1% in the LASSO regression analyses. All data by the primary parent.

[b] Measured at the third wave (after one year). Stigma was retrospectively assessed as perceived during the past year while being in care at the outpatient clinic. All other measures assessed at baseline.

[c] Data from primary parent (i.e., who has most parenting time with the child) and secondary parent.

Abbreviations: Ref. = reference category, PPSSS = modified version of the Parents' Perceived Stigma of Service Seeking Scale (Williams and Polaha, 2014), SDQ = Strength and Difficulties Questionnaire (Goodman, 1997), MHI = Mental Health Index-5 (Berwick et al., 1991), ASRS = ADHD Self-Report Scale (Kessler et al., 2005), APQ = Alabama Parenting Questionnaire (Shelton et al., 1996; Essau et al., 2006), PSS = Parental Stress Scale (Berry and Jones, 1995), VGFO = Parental Questionnaire Family Functioning (Veerman et al. 2012), CEQ = Credibility and Expectancy Questionnaire (Nock et al., 2007), PMI = Parental Motivation Inventory (Nock and Photos, 2006).

References

1. Williams SL, Polaha J. Rural parents' perceived stigma of seeking mental health services for their children: Development and evaluation of a new instrument. *Psychol Assess*. 2014;26(3):763-773. doi:10.1037/a0036571
2. Frick PJ. *Inventory of Callous-Unemotional Traits*. University of New Orleans; 2004.
3. Roose A, Bijttebier P, Decoene S, Claes L, Frick PJ. Assessing the affective features of psychopathy in adolescence: a further validation of the inventory of callous and unemotional traits. *Assessment*. 2010;17(1):44-57. doi:10.1177/1073191109344153
4. Goodman R. The Strengths and Difficulties Questionnaire: a research note. *J Child Psychol Psychiatry*. 1997;38(5):581-586. doi:10.1111/j.1469-7610.1997.tb01545.x
5. Berwick DM, Murphy JM, Goldman PA, Ware JE, Barsky AJ, Weinstein MC. Performance of a five-item mental health screening test. *Med Care*. 1991;29(2):169-176. doi:10.1097/00005650-199102000-00008
6. Adler LA, Spencer T, Faraone S V., et al. Validity of pilot adult ADHD Self-Report Scale (ASRS) to rate adult ADHD symptoms. *Ann Clin Psychiatry*. 2006;18(3):145-148. doi:10.1080/10401230600801077
7. Kessler RC, Adler L, Ames M, et al. The World Health Organization adult ADHD self-report scale (ASRS): A short screening scale for use in the general population. *Psychol Med*. 2005;35(2):245-256. doi:10.1017/S0033291704002892
8. Shelton, K.K., Frick, P.J., & Wootton, J. (1996). Assessment of parenting practices in families of elementary school-age chil dren. Journal of Clinical Child Psychology, 25(3), 317–329. doi:10.1207/s15374424jccp2503_8
9. Essau, CA, Sasagawa, S, & Frick, PJ (2006). Psychometric properties of the Alabama Parenting Questionnaire. Journal of Child and Family Studies, 15, 597-616. doi:10.1007/s10826-006-9036-y
10. Van Lier PAC., Crijnen AAM. *Alabama Parenting Questionnaire, Nederlandse Vertaling [Alabama Parenting Questionnaire, Dutch Translation]*. Erasmus MC: Unpublished manuscript; 1999.
11. Johnston C, Mash EJ. A measure of parenting satisfaction and efficacy. *J Clin Child Psychol*. 1989;18(2):167-175. doi:10.1207/s15374424jccp1802_8
12. Berry JO, Jones WH. The parental stress scale: Initial psychometric evidence. *J Soc Pers Relat*. 1995;12(3):463-472. doi:0803973233
13. Veerman, J.W., Janssen, J., Kroes, G., De Meyer, R., Nguyen, L., & Vermulst, A. (2012). Vragenlijst Gezinsfunctioneren volgens Ouders (VGFO). Handleiding. Nijmegen: Praktikon.Borkovec TD, Nau SD. Credibility of analogue therapy rationales. *J Behav Ther Exp Psychiatry*. 1972;3(4):257-260. doi:10.1016/0005-7916(72)90045-6
14. Nock MK, Ferriter C, Holmberg E (2007) Parent beliefs about treatment credibility and effectiveness: assessment and relation to subsequent treatment participation. J Child Fam Stud 16(1):27– 38. https ://doi.org/10.1007/s1082 6-006-9064-7
15. Nock MK, Photos V. Parent motivation to participate in treatment: Assessment and prediction of subsequent participation. *J Child Fam Stud*. 2006;15(3):345-358. doi:10.1007/s10826-006-9022-4
16. Ganzeboom HBG, De Graaf PM, Treiman DJ. A standard international socio-economic index of occupational status. *Soc Sci Res*. 1992;21(1):1-56. doi:10.1016/0049-089X(92)90017-B

17. Sampson RJ, Graif C. Neighborhood social capital as differential social organization: Resident and leadership dimensions. *Am Behav Sci*. 2009;52(11):1579-1605. doi:10.1177/0002764209331527
